# Hospital Notes

**Published:** 1871-11

**Authors:** C. C. F. Gay

**Affiliations:** Surgeon to the Buffalo General Hospital


ART. II.—*Hospital Notes*, by C. C. F. GAY, M.D., Surgeon to the Buffalo General Hospital.

———

*Varicose Ulcers.* Case 1.—B. H., aged 60 years, has an ulcer upon right leg near ankle, of two years standing, measuring two inches in length by one inch in breadth. The ulcer is caused by varicose condition of the veins of the leg.

The remedy that suggests itself for the cure of the ulcer is the remedy for the cure of the varicose veins. Five eschars were made with the potassa cum calce, poultices were applied, and in six weeks the patient was discharged, well. The ulcer healed simultaneously with the healing of the eschars.

Case 2.—Mrs A. B., aged 39 years, has two ulcers, the largest measuring three-quarters of an inch by two inches. These ulcers are the result of varicose veins, and will not heal until the veins are obliterated by the caustic potass. The ulcers have existed for a year or longer, and are increasing in size. She has resorted to treatment by poultices and lotions without benefit. On April 1st, 1871, five eschars were made. and in three weeks time, when the eschars were healed, the ulcers were also healed, and covered, apparently with as healthy integument as that contiguous to it.

Case 3.—Mrs O., aged 55 years. This patient has ulcers at ankle joint, one of which is two inches in diameter. She has also varicose veins of both legs of long standing, she thinks since childhood. On June 3d the potassa cum calce was applied for the obliteration of the veins. Eight eschars upon the left leg and five upon the right leg.

The caustic was used in the following manner: Adhesive strips, through each of which a hole was made the size of a small pea, were applied directly over the vein to be obliterated. The potassa cum calce, pulverized, was added to just enough alcohol to make a paste. This paste was applied to the vein through the small opening made in the adhesive strap, and allowed to remain twenty minutes, when it was removed, and the surface washed with vinegar. The after treatment consisted simply of slippery elm poultices. One week after eschars were made, simple erysipelas attacked both limbs. This disease was prevalent in the hospital at the time; but on the 30th June I have noted that the ulcers are healed.

Thus it happens that eschars produced in the manner described accomplishes a two-fold object at once, the cure of varicose veins and the cure of varicose ulcers. Of the several cases treated at the hospital there has not been a failure in a single instance, and in no other case has there been any dangerous complication, such as intercurrent erysipelas or phlebitis. Eight eschars upon one limb are, perhaps, too many for safety. I think it would be better to use but five, and repeat the operation if this number did not seem sufficient to obliterate all the veins requiring obliteration.

*Strangulated Inguinal Hernia—Operation—Death.*

Mr. G., aged 24 years, entered hospital May 16th. In the afternoon of that date Dr. Miner had visited him at his room and attempted to reduce an inguinal hernia by taxis. Failing in this, he divided the stricture external to the sac, which he observed permitted reduction of the tumor, but that action of abdominal muscles caused partial return. The patient was now sent to the Hospital. At my visit next morning, found the patient suffering greatly with pain, he was vomiting, had fever, and the scrotum much distended by the hernial sac. Chloroform was administered, and a failure made to reduce the hernia by taxis; cold applications were ordered, and morph. hypodermically. I immediately called together members of the staff for consultation, the result of which was to do nothing further at present.

On May 20th, there was erysipelas slightly diffused over the abdomen,vomiting and pain with febrile action. On consultation again with members of the hospital staff it was thought possible, from continuance of the symptoms that stricture might be found in the neck of the sac itself; and, assisted by Dr. Miner, I re-opened the wound, and on opening into the sac a large quantity of serum escaped. Tracing down with the finger, I found as had been anticipated, besides constriction of the ring, contraction of the neck of the sac. This was fully relieved by pressure of the finger and knife, but after all, the intestine could not be returned. Upon further examination the intestine was found everywhere adherent to the sac, and the sac to the surrounding parts, so that it could not be fully returned. These adhesions were with difficulty broken up with the finger, when the protruding bowel was made to return within the abdominal cavity, and did not again escape. The patient succumbed on the 23d, three days after the operation.

*Operation for Radical Cure of Inguinal Hernia.*

S. M., aged 8 years, hernia of right side, is congenital. On April 15th, 1871, assisted by Dr. Harrington, house physician, I operated, although the case for operation was very unpromising. Before beginning the operation I stated that it might not be completed on account of the apparent absence of a firm inferior pillar; it felt to the finger like a soft portion of protruding integument, therefore, I thought when the needle penetrated it that it would tear through the margin, or that there would not be sufficient strength in the pillar after the silver wire had been introduced to enable me to draw the superior and inferior pillars in apposition. However, the operation was satisfactorily completed, and gave good promise of radical cure. On May 9th the patient was allowed to get up and run about the wards. The patient was examined daily and not the slightest protrusion was discoverable until the 21st when I was showing the case to members of the staff. I saw for the first time some degree of tumefaction, which, on examination, proved to be the bowel again making its way through the ring. Undoubtedly the failure of the case was entirely owing to the abnormal condition of the interior pillar described above. Whether or not the bowel will again work its way and descend into the scrotum is not yet determined, but it is to be presumed that the bowel will again descend unless prevented by the application of a truss. The ring is partially closed. It is to be regretted that a second ligature or suture, had not been employed ; undoubtedly two sutures would have maintained the pillars in apposition, and in time, say six or eight months, the ring would have been permanently closed. A truss may now be used with much more hope of ultimate success, and will accomplish its object in much less time with the aid of the operation than without it.

